# Sars-Cov-2 e Injúria Miocárdica com Supradesnivelamento de ST sem Doença Coronariana: Relato de Caso e Breve Revisão da Literatura

**DOI:** 10.36660/abc.20201268

**Published:** 2021-08-09

**Authors:** Carolina Ragonetti, Enzo Oku Martinazzo, Felipe Montesano Fazionato, Guilherme Osório Guimarães Ferreira, Milena Piccolo Santana, Camila Hartmann

**Affiliations:** 1 Pontifícia Universidade Católica do Paraná CuritibaPR Brasil Pontifícia Universidade Católica do Paraná, Curitiba, PR - Brasil; 2 Hospital Marcelino Champagnat CuritibaPR Brasil Hospital Marcelino Champagnat, Curitiba, PR - Brasil

**Keywords:** Pandemia, SARS-CoV-2, Coronavirus-19, Miocardite/complicações, Eletrocardiografia/métodos, Cardiomiopatia de Takotsubo, Angiografia Coronária/métodos

## Introdução

Durante a pandemia de SARS-CoV-2, há relatos de infecção cursando com injúria miocárdica aguda (IMA), que causa piores resultados clínicos. A manifestação pode ser com elevação de troponina, alterações de imagem e eletrocardiográficas.^1^ Nesse sentido, a IMA com supradesnivelamento de ST (SST) tem sido observada em alguns pacientes. Contudo, apesar de alteração isquêmica ao eletrocardiograma (ECG), os exames podem não evidenciar obstrução, não sendo oclusão coronariana a causa da injúria.^2^

## Relato de Caso

Masculino, 42 anos de idade, sem comorbidades prévias, admitido em hospital de Curitiba-PR com queixa de tosse seca há 6 dias e odinofagia há 2 dias, com piora no dia anterior, apresentando tosse com secreção amarelada, dispneia, mal-estar, febrícula, mialgia e cefaleia. Contato recente com pacientes positivos para SARS-CoV-2. Exame físico: bom estado geral, corado, hidratado, eupneico, FC 100bpm, FR 18mrpm, SpO_2_ 98%, temperatura 36,2°C e PA 226/158mmHg. Ausculta pulmonar com crepitantes no terço inferior de hemitórax esquerdo (HTE) e cardíaca sem alterações. Controle da pressão arterial (PA) feito com nitroglicerina e solicitação de exames.

**Figura 1:**
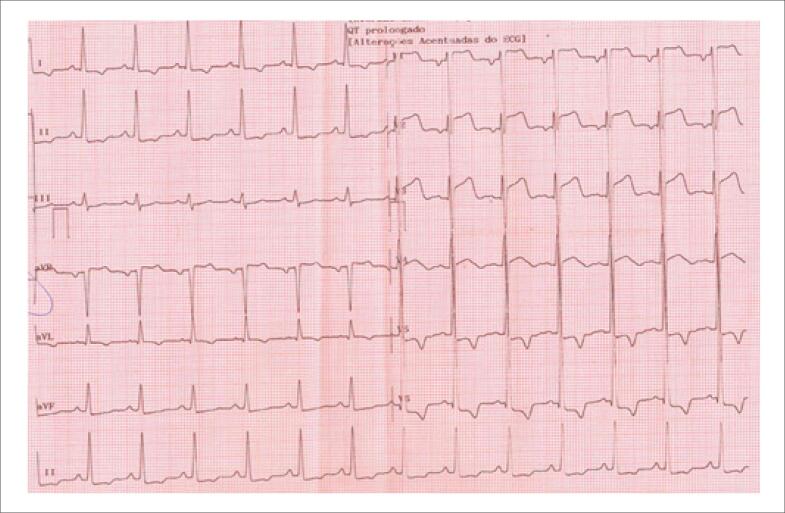
Eletrocardiograma de admissão.

Diante de troponina I 76,1pg/mL (VR<2,3pg/mL), foi realizado ECG (Figura 1), evidenciando ritmo sinusal, SST em V1 a V3 e sobrecarga de ventrículo esquerdo (VE). Paciente relatou que, na noite anterior, houve episódios de dor em pontada em HTE de poucos minutos.

**Figura 2:**
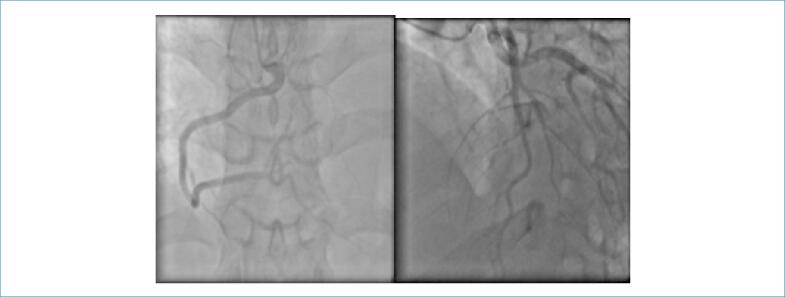
Cineangiocoronariografia de artéria coronária direita e artéria descendente anterior.

A cineangiocoronariografia (Figura 2) evidenciou disfunção segmentar de VE e ausência de trombos ou processo aterosclerótico significativo em coronárias.

No D2, já na UTI, foi iniciado hidralazina, nitrato, anlodipino e carvedilol para desmame da nitroglicerina e controle da PA. Foi necessário uso de cateter de O_2_ nasal 3L/min e início de ceftriaxona, azitromicina e dexametasona.

Tomografia: opacidades pulmonares consolidativas, broncograma aéreo e vidro fosco periférico associados a densificações subpleurais no lobo inferior esquerdo. Ecocardiograma: VE aumentado com padrão de hipertrofia concêntrica importante e função sistólica moderadamente comprometida. Átrio esquerdo (AE) aumentado, discreto refluxo mitral, tricúspide e aórtico e ectasia da raiz da aorta.

**Figura 3:**
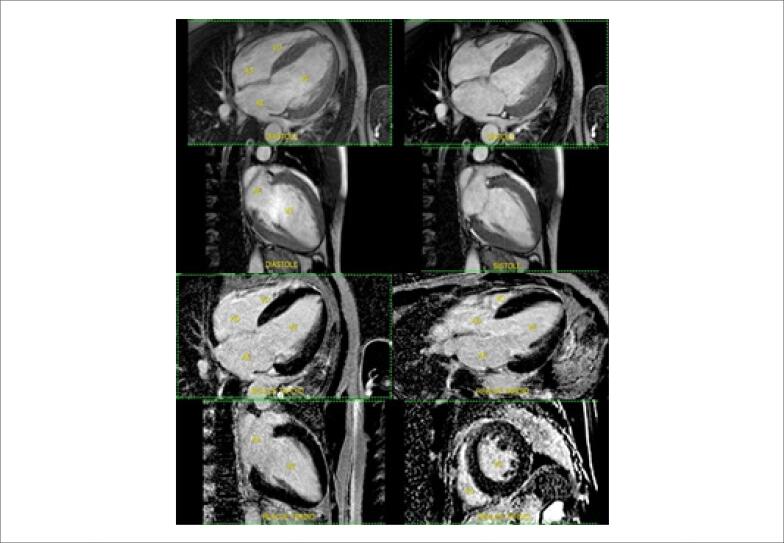
Ressonância magnética cardíaca com estudo dinâmico (superior) e realce tardio (inferior).

RT-PCR positivo para SARS-CoV-2. Levantadas hipóteses diagnósticas de miocardite associada ao SARS-CoV-2, trombose com lise espontânea, lesão microvascular, insuficiência cardíaca (IC) por miocardiopatia hipertensiva ou de Takotsubo. Recebeu alta com tratamento otimizado para IC. Em retorno após 60 dias, RNM cardíaca (Figura 3) evidenciou: dilatação da cavidade de VE associada a disfunção sistólica global importante (FEVE 23%), dilatação da cavidade do ventrículo direito associada a disfunção sistólica global leve (FEVD 43%), hipertrofia excêntrica do VE, AE dilatado e ausência de necrose.

## Discussão

Neste relato, a IMA, evidenciada por SST e troponina elevada, pode ter diversas explicações: uma das hipóteses é a ocorrência de lesão miocárdica direta pelo vírus (miocardite). Contudo, a RM não demonstrou padrão de fibrose mesocárdica, edema ou necrose, não corroborando essa hipótese. Pelo fato de o diagnóstico ter sido tardio e a cineangiocoronariografia não ter evidenciado trombos ou processo aterosclerótico, outra possibilidade é ocorrência de trombose com lise espontânea ou lesão microvascular – a hipercoagulabilidade vista no estado pró-inflamatório na Covid-19 predispõe eventos coronarianos agudos.^1^^,^^3^ Outra hipótese é cardiomiopatia Takotsubo-*like*, que pode ocorrer em pacientes com SARS-CoV-2.^4^ Entretanto, a ventriculografia realizada com a cineangiocoronariografia e o ecocardiograma não evidenciaram padrão dessa cardiomiopatia, que poderia ser excluída caso a miocardite fosse confirmada.^5^ Por fim, há possibilidade de associação de alguma hipótese anterior com cardiomiopatia hipertensiva: apresentou pico hipertensivo e provável hipertensão não diagnosticada.

Quanto à fisiopatologia da Covid-19, há a ligação da proteína *spike* do vírus ao receptor da ECA-2, após a TMPRSS2 ativar a *spike*.^3^ Então, ocorre entrada do SARS-CoV-2 nas células através da ECA-2, presente em múltiplos tecidos do organismo, incluindo cardiomiócitos. Essa enzima converte angiotensina II, um componente inflamatório, vasoconstritor, oxidativo e fibrótico, em angiotensina (1-7) , de ações opostas. Portanto, ocorrem duas principais situações: entrada do vírus em células miocárdicas e, uma vez que os receptores estão bloqueados por proteínas virais, acúmulo de angiotensina II, além de liberação massiva de citocinas.^6^^–^^8^

Além disso, estudos mostram que a IMA pode ocorrer na Covid-19 por isquemia miocárdica ou processo não isquêmico. A lesão é relacionada a quadros mais graves da doença, como desenvolvimento de IC em até 23% dos pacientes.^9^ Na China, estudos mostram que até 17% dos pacientes apresentaram elevação da troponina.^7^^,^^8^

A elevação de troponina em quadro não isquêmico de injúria miocárdica pode ser explicada por hipóxia tecidual, sepse, resposta inflamatória sistêmica, tromboembolismo venoso e estresse miocárdico.^8^ Havendo obstrução, a hipótese é que o vírus possa causar instabilidade e hemorragias intraplaca, gerando exposição do colágeno, lesão microvascular e formação de trombos.^1^^,^^3^^,^^8^ Na ausência de processo aterosclerótico, acredita-se que o desbalanço entre oferta e consumo de oxigênio cause um infarto agudo do miocárdio (IAM) tipo 2.^3^ Além dos mecanismos de lesão miocárdica diretos, há mecanismos indiretos: tempestade de citocinas e Takotsubo. Essa cardiomiopatia representa até 3% das suspeitas de síndrome coronariana aguda, e sabe-se que situações de infecção respiratória, estresse emocional e físico podem ser gatilhos, cursando com disfunção transitória do VE.^3^^,^^5^^,^^7^

Comparando com casos semelhantes (Tabela 1), Aragão et al.,^2^ descreveram elevação de troponina, mas difere do nosso paciente pela ausência de IC constatada pela redução significativa da fração de ejeção do ventrículo esquerdo (FEVE). Inciardi et al.,^10^ também descreveram redução da FEVE, porém, mais branda. Já, Huyut^11^ não mostra elevação da troponina, mas a redução transitória da FEVE sugere ocorrência de miocardiopatia.^11^

**Tabela 1:** Comparação de Casos

Casos	Curitiba	Aragão et al.^2^	Inciardi et al.^10^	Huyut^11^
Idade/sexo	42/masculino	39/masculino	53/feminino	59/feminino
FEVE	23%	62%	40%	52%
Troponina I	76,1pg/mL	25,20ng/mL	0,89*	Normal
ECG	SST	SST	SST	Sem alteração
RT-PCR SARS-CoV-2	Positivo	Positivo	Positivo	Positivo
Hipocinesia	Difusa	Segmento médio parede anterosseptal	Difusa	–

* Troponina T ultrassensível. FEVE: fração de ejeção do ventrículo esquerdo; ECG: eletrocardiograma.

Stefanini et al.,^12^ demostraram que 85,7% dos pacientes de uma série de casos tinham sinais de infarto com SST como primeira manifestação sintomática da Covid-19, e que 39,3% não apresentavam doença obstrutiva. Nosso paciente teve SST, mas não como primeira manifestação, além de não ter apresentado oclusão na cineangiocoronariografia. Assim como a maioria dos pacientes do estudo, o nosso seguiu padrão de prognóstico benigno.^12^

## Conclusão

Neste relato, apresentamos um caso atípico de manifestação cardiológica da Covid-19, em que houve SST sem evidência de coronariopatia, evoluindo para IC com fração de ejeção reduzida. Como discutido, as hipóteses de miocardite viral, trombose com lise espontânea, lesão microvascular, Takotsubo e cardiomiopatia hipertensiva não foram completamente estabelecidas, podendo até coexistir. Por fim, enaltecemos que o esclarecimento dos mecanismos envolvidos visa à identificação precoce e ao manejo adequado dos pacientes, acarretando melhora dos desfechos e compreensão das possíveis sequelas.
