# Supplementary material for: Sars-Cov-2 e Injúria Miocárdica com Supradesnivelamento de ST sem Doença Coronariana: Relato de Caso e Breve Revisão da Literatura
Source: Arq Bras Cardiol. 2021 Aug 9;117(2):411–4. [Article in Portuguese] doi: 10.36660/abc.20201268 (PMC8395782; doi:10.36660/abc.20201268)
Supplement: Translation

# Translation: SARS-CoV-2 and Myocardical Injury with ST-Elevation without Coronary Disease

**Authors:** Carolina Ragonetti, Enzo Oku Martinazzo, Felipe Montesano Fazionato, Guilherme Osório Guimarães Ferreira, Milena Piccolo Santana, Camila Hartmann

**Affiliations:** 1 CuritibaPR Brazil Pontifícia Universidade Católica do Paraná, Curitiba, PR - Brazil.; 2 CuritibaPR Brazil Hospital Marcelino Champagnat, Curitiba, PR - Brazil.

**Keywords:** Pandemics, Coronavirus-19, SARS-CoV-2, Myocarditis/complications, Electrocardiography/methods, Takotsubo Cardiomyopathy, Coronary Angiography/methods

## Introduction

Throughout the SARS-CoV-2 pandemic, there have been reports of infection leading to acute myocardial injury, causing worse clinical outcomes. The manifestations can include troponin elevation, imaging exam abnormalities and electrocardiographic changes.^1^ Accordingly, ST-segment elevation (STE) acute myocardial injury has been observed in some patients. However, despite the electrocardiogram (ECG) ischemic changes, complementary exams may not show any obstruction, excluding coronary occlusion as the cause of the injury.^2^

## Case Report

A 42 year-old male patient, with no previous comorbidities, was admitted to a hospital in Curitiba, state of Paraná, Brazil, complaining of nonproductive cough for 6 days and odynophagia for 2 days, with symptom worsening on the previous day, including cough with yellowish sputum, dyspnea, malaise, fever, myalgia and headache. He reported recent contact with SARS-CoV-2 positive patients. On physical examination, he looked well, awake, alert, oriented, hydrated, eupneic, with a pulse of 100bpm, RR 18 breaths per minute, SpO_2_ 98%, temperature 36.2°C and BP 226/158mmHg. Pulmonary auscultation disclosed crackling rales in the lower third of the left hemithorax and the cardiovascular examination showed no abnormalities. BP control was achieved with Nitroglycerine and laboratory tests were requested.

On account of troponin I level at 76.1pg/mL (RV<2.3 pg/mL), an ECG was performed (Figure 1), showing sinus rhythm, STE from V1 to V3 and LV hypertrophy. The patient reported that he experienced episodes of stinging pain in left hemithorax on the previous night, lasting a few minutes.

**Figure 1:** ECG performed upon admission.

The coronary artery angiography (figure 2) showed segmental LV dysfunction and absence of thrombi or any significant atherosclerotic process in the coronary arteries.

**Figure 2:** Right coronary and left anterior descending arteries coronary artery angiography.

On day 2, in the ICU, Hydralazine, Nitrate, Amlodipine and Carvedilol were administered for Nitroglycerin weaning and BP control. The use of a nasal O_2_ catheter 3L/min was required and Ceftriaxone, Azithromycin and Dexamethasone were initiated.

The CT showed pulmonary opacities, suggesting lung consolidation, air bronchogram and peripheral ground-glass opacity associated with subpleural densifications in the left lower lobe. The echocardiogram showed LV enlargement with a significant concentric hypertrophy pattern and moderate systolic dysfunction. Left atrial enlargement, mild mitral, tricuspid and aortic regurgitation and aortic root ectasia were observed.

A positive RT-PCR result for SARS-CoV-2 was obtained. Diagnostic hypotheses were raised for myocarditis associated with SARS-CoV-2, thrombosis with spontaneous lysis, microvascular injury, heart failure (HF) due to hypertensive or Takotsubo cardiomyopathy. The patient was discharged with optimized HF treatment. Upon return after 60 days, cardiac MRI (figure 3) showed: LV dilation associated with significant global systolic dysfunction (LVEF 23%), RV dilation associated with mild global systolic dysfunction (RVEF 43%), eccentric LV hypertrophy, left atrial dilation and absence of myocardial necrosis.

**Figure 3:** Dynamic contrast-enhanced MRI (top) and delayed enhancement (bottom).

## Discussion

In this case report, acute myocardial injury, evidenced by STE and elevated troponin, may lead to several hypothesis: the occurrence of direct myocardial injury by the virus (myocarditis). However, the MRI did not show a pattern of mesocardial fibrosis, edema or necrosis, which does not corroborate the former proposition. Because of the late diagnosis and the fact that the coronary artery angiography did not show thrombi or any atherosclerotic process, another possibility is the occurrence of thrombosis with spontaneous lysis or microvascular injury – as the hypercoagulability seen in the pro-inflammatory state in COVID-19 predisposes to acute coronary events.^1^^,^^3^ Another proposition is the Takotsubo-like cardiomyopathy, which can occur in patients with SARS-CoV-2.^4^ However, a cardiac ventriculography performed with coronary artery angiography and echocardiogram did not show a pattern compatible with this cardiomyopathy, which could also be excluded if the myocarditis was confirmed.^5^ Finally, there is the possibility of association of some of the previous hypotheses with hypertensive cardiomyopathy, as the patient showed a hypertensive peak and probably had undiagnosed hypertension.

As for the pathophysiology of the COVID-19, there is the binding of the virus’s spike protein to the ACE-2 receptor, after spike activation by TMPRSS2.^3^ Then, SARS-CoV-2 enters the cells through ACE-2 receptor, present in multiple body tissues, including cardiomyocytes. This enzyme converts angiotensin II, an inflammatory, vasoconstrictor, oxidative and fibrotic component, into angiotensin (1-7), with contrasting effects. Therefore, two main situations occur: the virus enters myocardial cells and, as the receptors are blocked by viral proteins, there is an increase in angiotensin II, in addition to a massive release of cytokines.^6^^–^^8^

Studies also show that acute myocardial injury can occur in COVID-19 due to myocardial ischemia or a non-ischemic process. The injury is related to more severe conditions of the disease, such as the development of HF in up to 23% of patients.^9^ In China, studies suggest that up to 17% of COVID patients had elevated troponin levels.^7^^,^^8^

Troponin elevation in non-ischemic myocardial injury can be explained by tissue hypoxia, sepsis, systemic inflammatory response, venous thromboembolism and myocardial stress.^8^ If there is an obstruction, the hypothesis is that the virus may cause instability and intraplaque hemorrhage, exposing collagen, causing microvascular injury and thrombus formation.^1^^,^^3^^,^^8^ In the absence of the atherosclerotic process, it is possible that the imbalance between oxygen supply and demand results in a type 2 acute myocardial infarction.^3^ In addition to the direct myocardial injury mechanisms, there are indirect mechanisms: cytokine storm and Takotsubo. This cardiomyopathy represents almost 3% of acute coronary syndrome suspicions and it is known that conditions such as respiratory infection, emotional and physical stress can be triggers, leading to transient LV dysfunction.^3^^,^^5^^,^^7^

Compared with similar cases (Table 1), Aragão et al.^2^ described a troponin elevation, but it differs from our patient due to the absence of HF, verified by the significant reduction in the left ventricular ejection fraction (LVEF). Inciardi et al.^10^ also described an LVEF reduction; however, it was milder. Huyut,^11^ on the other hand, did not show an increase in troponin; however, the transient reduction in LVEF suggests cardiomyopathy.^11^

**Table 1:** Case Comparison

Cases	Curitiba	Aragão et al.^2^	Inciardi et al.^10^	Huyut^11^
Age/gender	42/male	39/male	53/female	59/female
LVEF	23%	62%	40%	52%
Troponin I	76.1pg/mL	25.20ng/mL	0.89*	Normal
ECG	STE	STE	STE	Normal
RT-PCR SARS-CoV-2	Positive	Positive	Positive	Positive
Hypokinesis	Diffuse	Mid-cavity anteroseptal segment	Diffuse	-

* High-sensitivity cardiac troponin-T. LVEF: left ventricular ejection fraction; ECG: electrocardiogram.

Stefanini et al.^12^ demonstrated that 85.7% of patients in a case series had signs of infarction with STE as the first symptomatic manifestation of COVID-19 and that 39.3% did not have any evidence of obstructive disease. Our patient had STE, but it was not the first manifestation, in addition to not showing occlusion in the coronary artery angiography. Like most of their patients, ours followed a benign pattern.^12^

## Conclusion

This case report described an atypical case of cardiac manifestation of COVID-19, in which there was STE without evidence of coronary disease, progressing to HF with reduced ejection fraction. As previously discussed, the hypotheses of viral myocarditis, thrombosis with spontaneous lysis, microvascular injury, Takotsubo and hypertensive cardiomyopathies have not been fully established, and may even coexist. Finally, we emphasize that the elucidation of the involved mechanisms contributes to the earlier identification and adequate management of patients, leading to better outcomes and understanding of possible sequelae.
